# Supplementary material for: Ultra-sensitive polarization-resolved black phosphorus homojunction photodetector defined by ferroelectric domains
Source: Nat Commun. 2022 Jun 9;13:3198. doi: 10.1038/s41467-022-30951-y (PMC9184637; doi:10.1038/s41467-022-30951-y)
Supplement: Supplementary file 1 — Supplementary Information [file 41467_2022_30951_MOESM1_ESM.pdf]

Supplementary Information for
Ultra-sensitive polarization-resolved black phosphorus homojunction photodetector defined by ferroelectric domains

Shuaiqin Wu,[1,2#] Yan Chen,[1#] Xudong Wang,[1*] Hanxue Jiao,[1,2] Qianru Zhao[1,2], Xinning Huang,[1,2] Xiaochi Tai,[1,2] Yong Zhou,[1] Hao Chen,[1,2] Xingjun Wang,[1] Shenyang Huang[3], Hugen Yan,[3] Tie Lin,[1,2] Hong Shen,[1,2] Weida Hu,[1,2] Xiangjian Meng,[1,2] Junhao Chu,[1,2] Jianlu Wang,[1,4,5*]

[1]State Key Laboratory of Infrared Physics, Shanghai Institute of Technical Physics, Chinese Academy of Sciences, No.500 Yutian Road, Shanghai 200083, China.

[2]University of Chinese Academy of Sciences, No.19 A Yuquan Road, Beijing 100049, China.

[3]State Key Laboratory of Surface Physics, Key Laboratory of Micro- and Nano-Photonic Structures (Ministry of Education), and Department of Physics, Fudan University, Shanghai 200433, China.

[4]Frontier Institute of Chip and System, Fudan University, Shanghai 200433, China.

[5]Shanghai Frontier Base of Intelligent Optoelectronics and Perception, Institute of Optoelectronics, Fudan University, Shanghai, 200433, China.

Contacts with:

Xudong Wang: wxd0130@mail.sitp.ac.cn

Jianlu Wang: jlwang@mail.sitp.ac.cn

[#]These authors contributed equally: Shuaiqin Wu, Yan Chen.

Table of contents

Supplementary Fig. 1 | The polarized Raman spectrum measurements........................ 3

Supplementary Fig. 2 | Polarization dependent infrared spectroscopy measurements.. 4

Supplementary Fig. 3 | The polarized Raman spectrum measurements........................ 5

Supplementary Fig. 4 | The remnant hysteretic field-dependent behavior of the PFM signal in 2-layer P(VDF-TrFE)... 6

Supplementary Fig. 5 | Stability and reliability of ferroelectric domains of PVDF-TrFE.. 7

Supplementary Fig. 6 | Electrical properties of BP at different P(VDF-TrFE) polarization state.. 8

Supplementary Fig. 7 | Polarization photoresponse of BP PN homojunction............... 9

Supplementary Fig. 8 | Polarization photoresponse of BP FET 10

Supplementary Fig. 9 | Polarization photoresponse of pristine BP FET with different wavelength.. 11

Supplementary Fig. 10 | Output and transfer curves of BP electrostatic doped through split gates 12

Supplementary Fig. 11 | Polarization photoresponse of BP PN homojunction defined by local electrostatic gating 13

Supplementary Fig. 12 | Output and transfer curves of BP electrostatic doped through split gates Spectral noise density of the device at 300 K and zero bias. 14

Supplementary Table 1 | The performance index comparison of polarization photodetectors based 2D materials 15

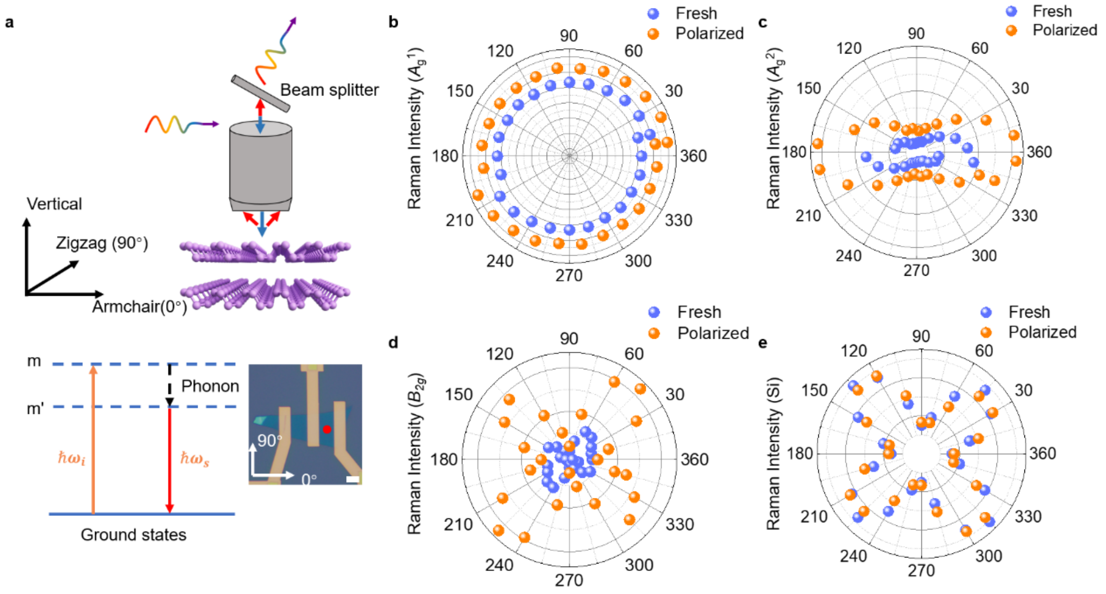

Supplementary Fig. 1 | The polarized Raman spectrum measurements. a. Schematic illustration of the Raman experiments and energy-level diagram for a Stokes Raman scattering process and optical image of BP. The red dot in the optical image represents the laser spot position. The crystal orientation is identified by Raman measurements. **b-e.** Polar plots of the peak intensities of A_g^1, A_g^2, B_{2g} and Si substrate before and after P(VDF-TrFE) polarized. The same Raman intensity of Si proves the experiment condition is unchanged. Scale bar is 5 μm.

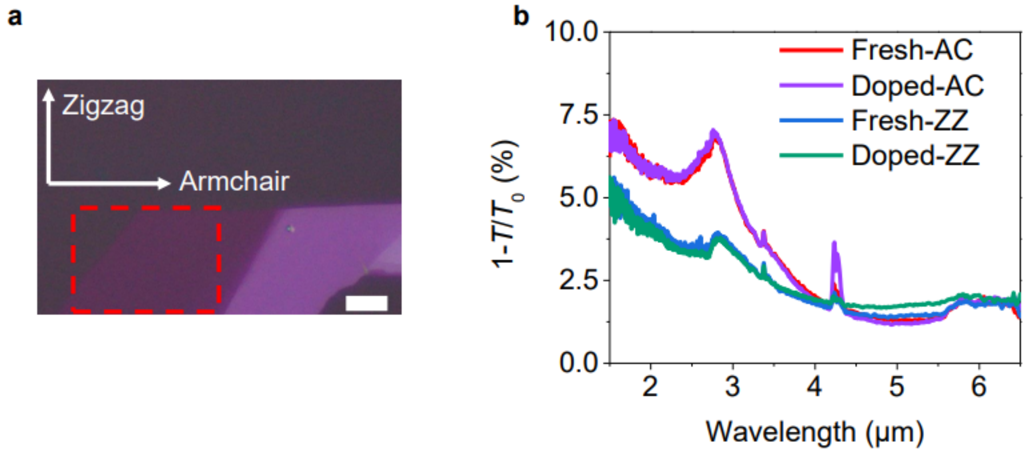

Supplementary Fig. 2 | Polarization dependent infrared spectroscopy measurements. a. Optical image of few-layer BP. The red dashed box represents the test area. **b.** Polarization-resolved infrared relative extinction spectra when light is polarized along the armchair (AC) direction and zigzag (ZZ) direction. Scale bar is 10 μm.

Polarization-resolved infrared spectroscopy was performed using a Bruker FTIR spectrometer (Vertex 70v) integrated with a Hyperion 2000 microscope. The light source is a combination of tungsten halogen lamp and globar, covering the wide energy range from mid-infrared to near-infrared.

BP was prepared by mechanical exfoliation method and transferred onto a Al_2O_3 substrate. After P(VDF-TrFE) covering whole sample by spin-coating method, the polarization-resolved infrared spectroscopy was performed (marked as Fresh state). Then, PFM was used to polarized P(VDF-TrFE) and polarization-resolved infrared spectroscopy was performed again at same condition (marked as Doped state). The absorption shows no obvious change before and after ferroelectric field doping in the armchair and zigzag direction.

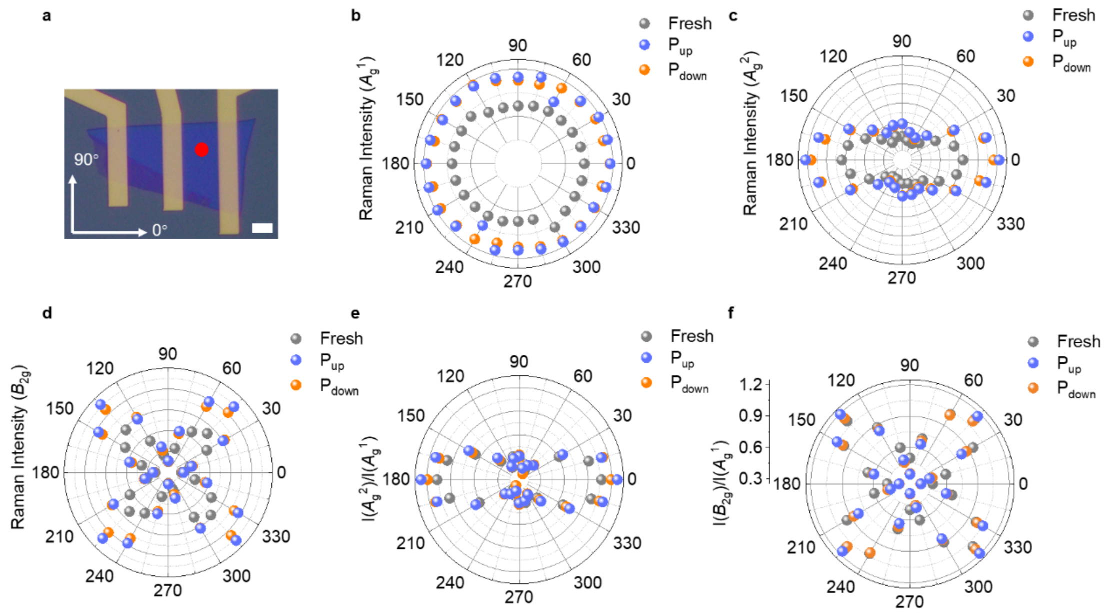

Supplementary Fig. 3 | The polarized Raman spectrum measurements. a. Optical image of the BP device. The red dot represents the laser spot. The crystal orientation is identified by Raman measurements. **b-f.** Polar plots of the peak intensities of A_g^1, A_g^2, B_{2g}, A_g^2/A_g^1 and B_{2g}/A_g^1 with three polarization state (Fresh state P_{up} state and P_{down} state). Scale bar is 5 μm.

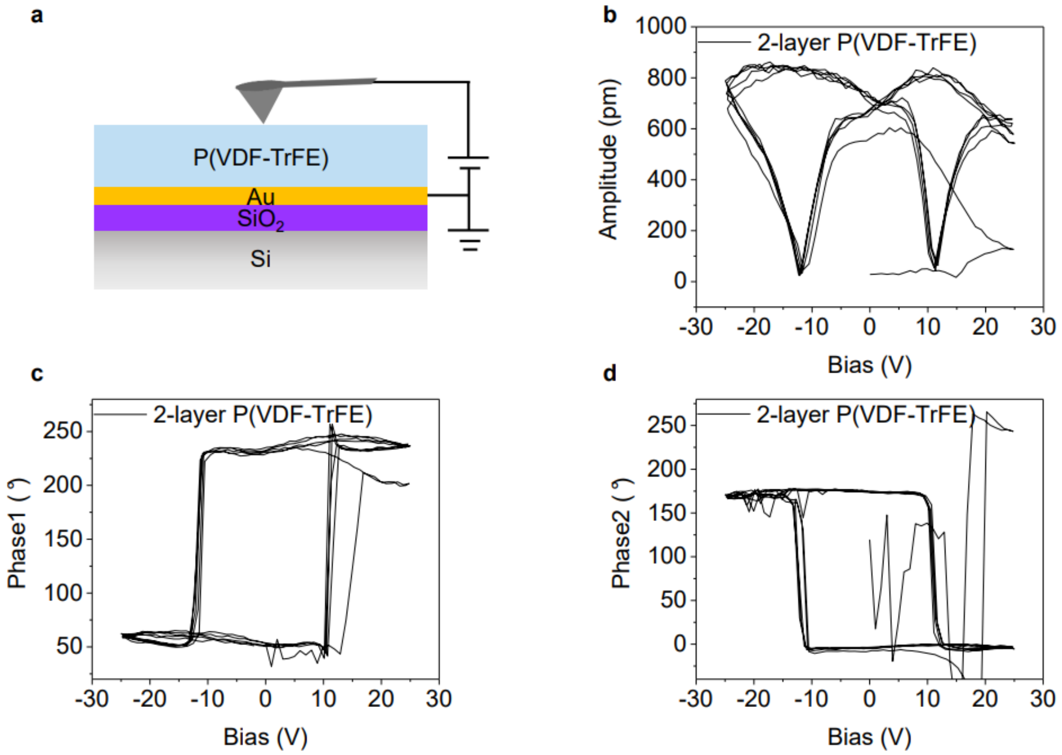

Supplementary Fig. 4 | The remnant hysteretic field-dependent behavior of the PFM signal in 2-layer P(VDF-TrFE). a. Schematic of the test setup. **b.** The amplitude hysteresis loops. **c-d.** The phase hysteresis loops from two channels.

Piezo Force Microscopy (PFM) can measure the mechanical response in ferroelectric materials when applying a voltage to the sample surface by a conductive AFM probe[1]. PFM technology is a non-destructive method to read and control of the nanodomains of ferroelectric materials[2]. By applying an electrical signal and recording the deformation signal of sample surface, we can obtain comprehensive information about the ferroelectrics. Here, we apply a voltage of ±25 V on the surface of P(VDF-TrFE) though a conductive probe and get the coercive voltage of 2-layer P(VDF-TrFE). Therefore, we can apply a voltage of -25 V on the left channel of BP and +25 V on the right channel to dope the left channel into p-type and right channel into n-type.

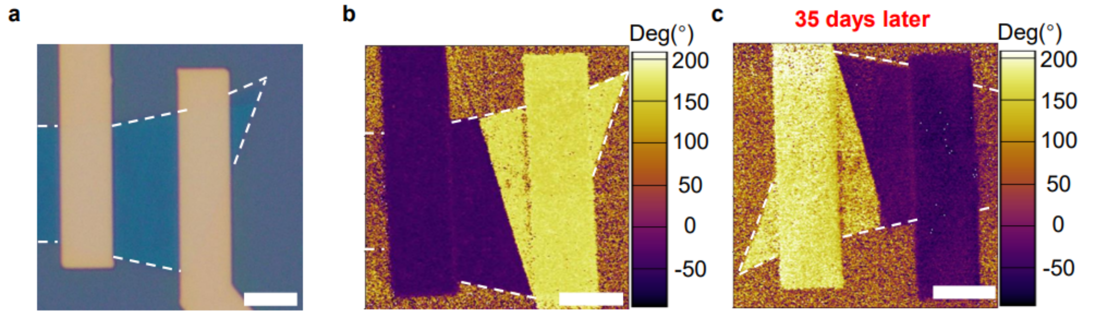

Supplementary Fig. 5 | Stability and reliability of ferroelectric domains of P(VDF-TrFE). a. Optical image of BP device covered by P(VDF-TrFE). Scale bar is 5 μm. **b**. PFM phase of P(VDF-TrFE) after polarized by PFM probes. Scale bar is 5 μm. **c.** Phase image after 35 days later. Scale bar is 5 μm.

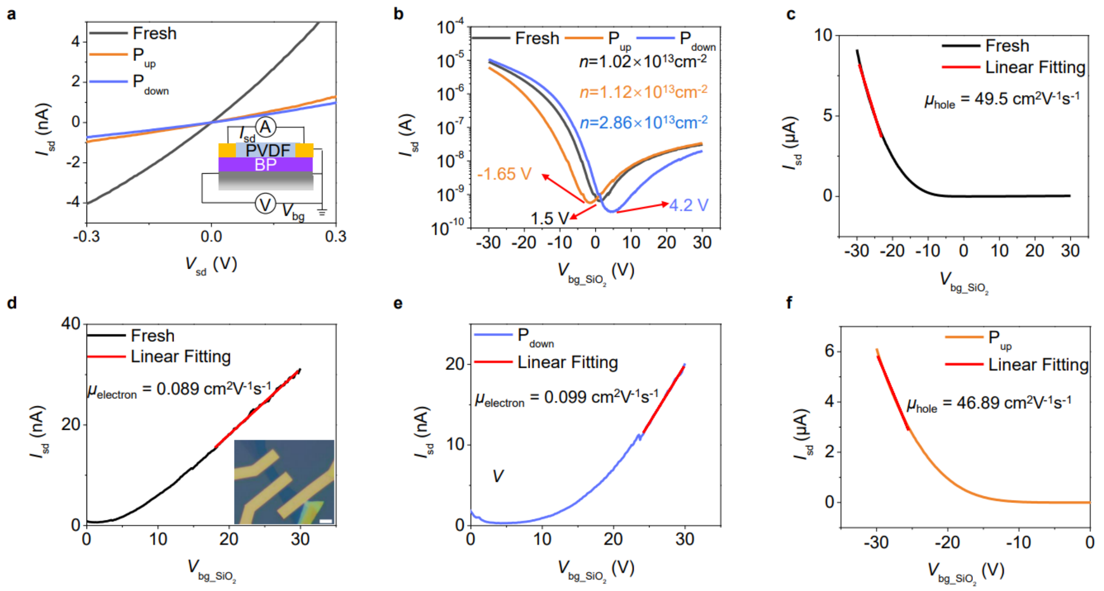

Supplementary Fig. 6 | Electrical properties of BP at different P(VDF-TrFE) polarization state. a. I_{sd}-V_{sd} curves at Fresh state P_{up} state and P_{down} state. **b.** Transfer curves at three different states with V_{sd} = 1 V. **c-d.** Hole mobility and electron mobility at fresh state, P_{up} state and P_{down} state, extracted from transfer curves in (**b**). Scale bar is 5 μm.

The total carrier density shown in (**b**) is calculated by n = (V_g-V_{ng}) × C_g/q, where V_g is the gate voltage at charge-neutrality point, V_{ng} = 0 V, C_g is the capacitance of 285 nm-SiO_2, q is electron charge[3]. The calculated carrier concentration in the up-polarized state and down-polarized state is 1.12 × 10^{13} cm^{-2} and 2.86 × 10^{13} cm^{-2}, respectively. The Debye length of the BP can be calculated by $L_D = \sqrt{\frac{\varepsilon_s kT}{q^2 N}}$, where the ε_s is the permittivity of BP, k is the Boltzmann's constant, q is the charge of electron, and N is the carrier concentration. The ε_s is calculated by the expression $\varepsilon_s = \varepsilon_r \varepsilon_0$, and ε_r is 5.76[4]. Therefor the calculated Debye lengths in the the up-polarized state and down-polarized state are 88 nm and 55 nm, respectively. The carrier mobility ($\mu_{electron}$ and μ_{hole}) of FET can be extracted by $\mu = \frac{L}{WC_iV_{sd}}(\frac{dI_{sd}}{dV_{bg}})$, where L and W are channel length and width, V_{sd} and V_{tg} are source-drain and top-gate voltage, dI_{sd}/dV_{tg} is extracted from the transfer curve in (**b**), C_i is the capacitance per area. The relative permittivity and thickness of SiO_2 are 3.5 and 285 nm, respectively.

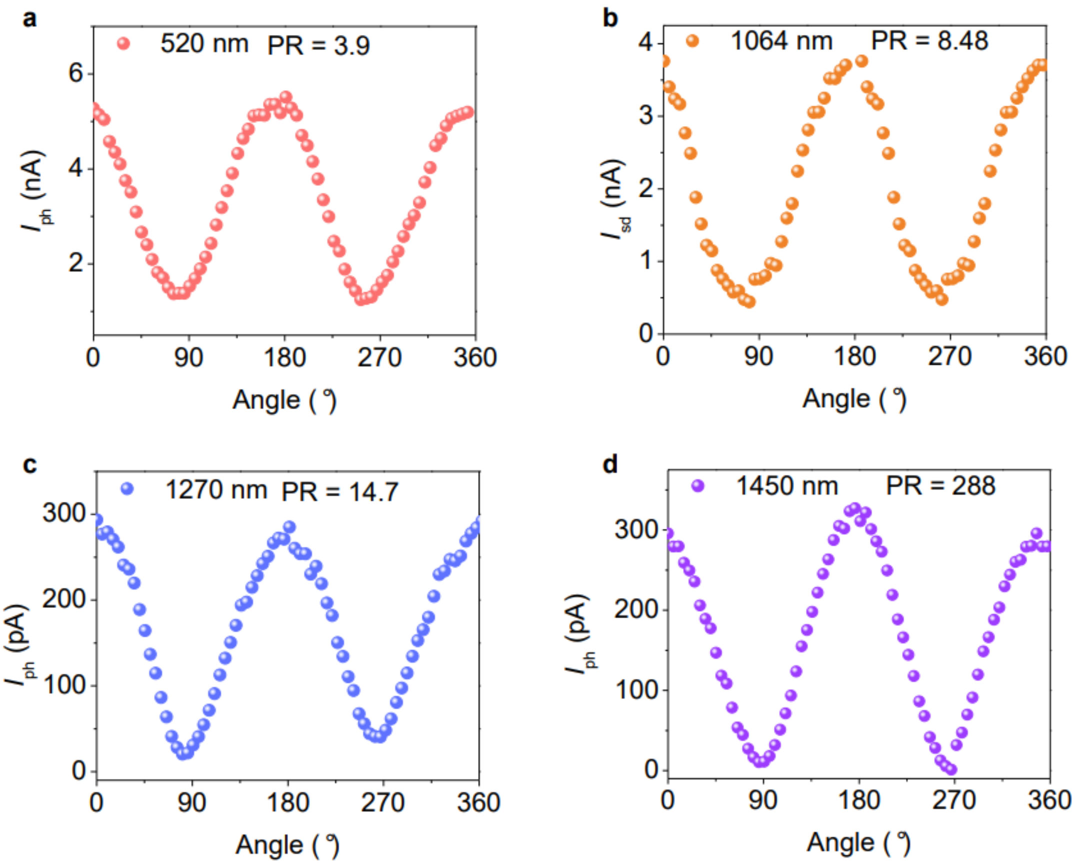

Supplementary Fig. 7 | Polarization photoresponse of BP PN homojunction. a-d. Photocurrent of the BP PN homojunction as a function of the polarization angle at 520 nm (10 μW),1064 nm (10 μW), 1270 nm (10 μW), and 1450 nm (10 μW).

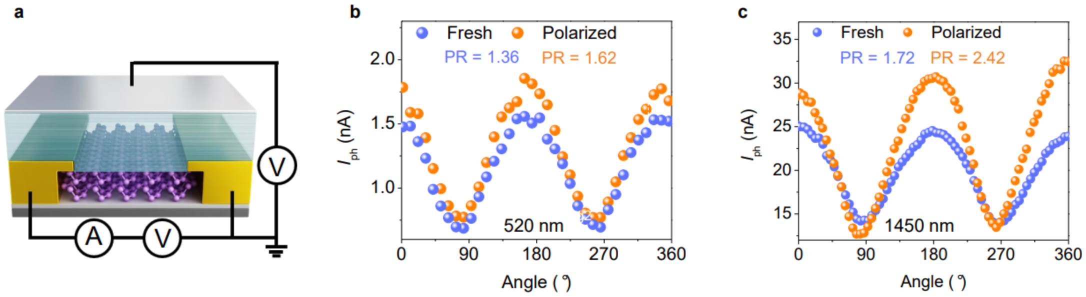

Supplementary Fig. 8 | Polarization photoresponse of BP FET. a. Structure schematic of BP FET. **b**, **c**. Photocurrent of the BP FET as a function of the polarization angle at 520 nm (10 μW) and 1450 nm (10 μW), V_{sd} = 0.2 V.

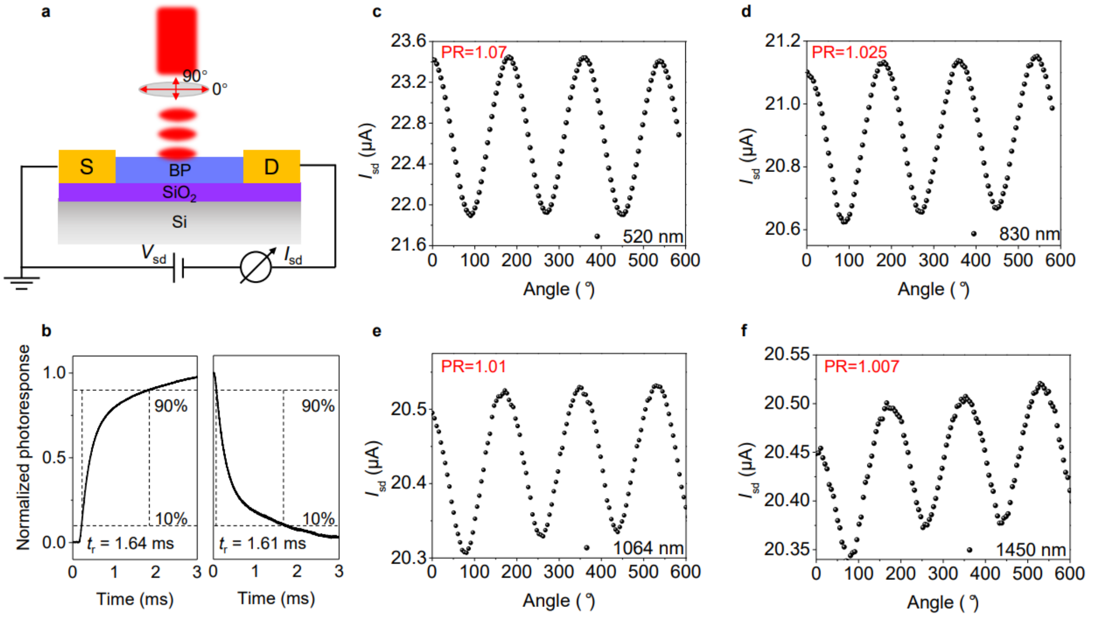

Supplementary Fig. 9 | Polarization photoresponse of pristine BP FET with different wavelength. a. Schematic of the test setup. b. 90-10% rise (t_r) and decay (t_f) time of photocurrent are measured as 1.64 ms and 1.61 ms, respectively. **c-f.** Photocurrent as a function of the incident light polarization angle at V_{sd} = 1 V. The wavelength varies from visible to near-infrared wavelength (520 nm, 830 nm, 1064 nm, 1450 nm).

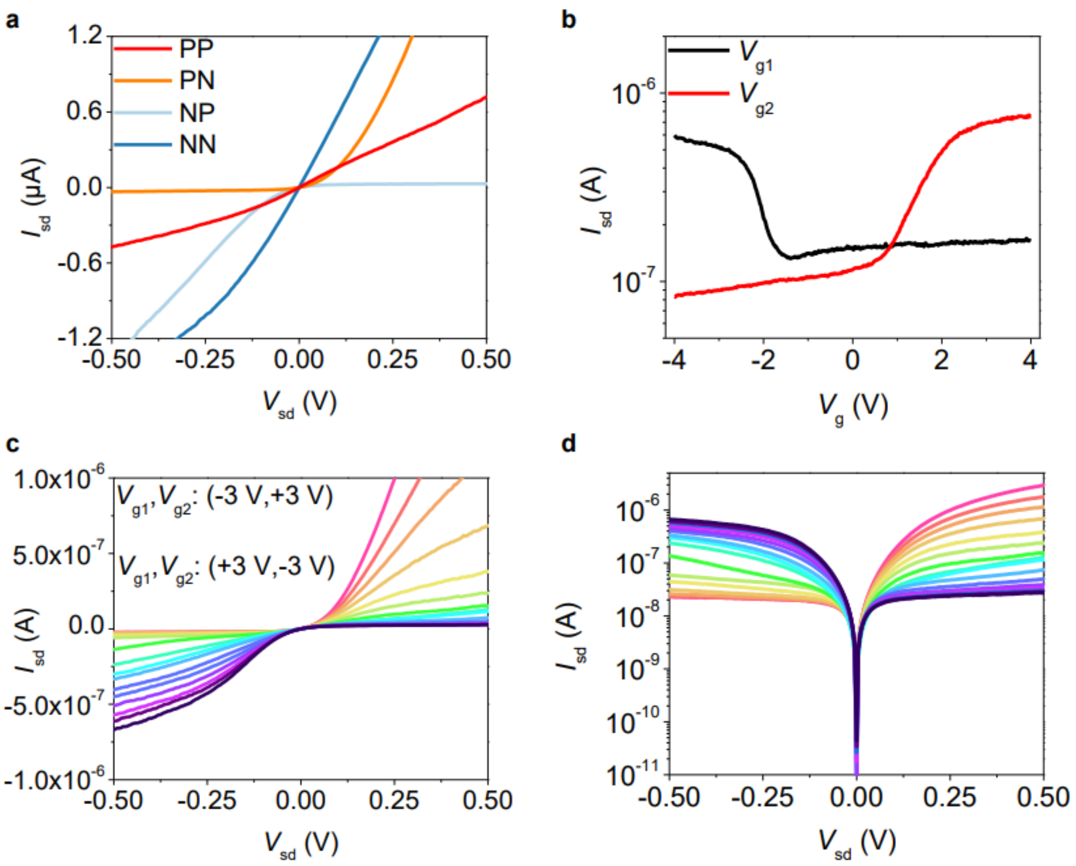

Supplementary Fig. 10 | Output and transfer curves of BP electrostatic doped through split gates. a. The output curve on a linear scale under different bottom gates voltage configuration (PP, PN, NP and NN). **b**. Transfer curves for V_{g1} and V_{g2} at V_{sd} = 0.5 V. **c**, **d**. The output curve plotted on linear and logarithmic scale with continuously changing gate voltage.

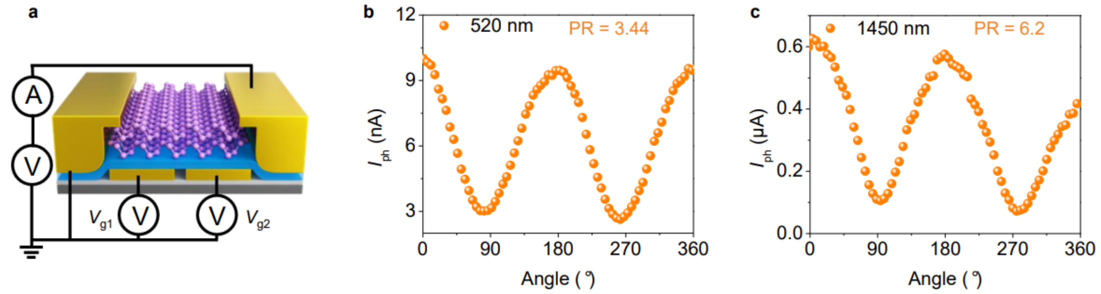

Supplementary Fig. 11 | Polarization photoresponse of BP PN homojunction defined by local electrostatic gating. a. Structure schematic of BP PN homojunction defined by local electrostatic gating. **b**, **c**. Photocurrent of the BP FET as a function of the polarization angle at 520 nm (10 μW) and 1450 nm (10 μW), V_{sd} = 0 V. The V_{g1} and V_{g2} are set to 3 V and -3 V, respectively.

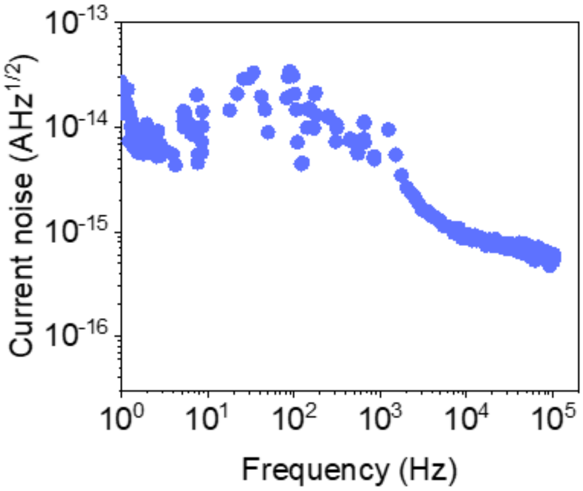

Supplementary Fig. 12 | Spectral noise density of the device at 300 K and zero bias.

Supplementary Table 1 | The performance index comparison of polarization photodetectors based 2D materials[5-17].

Type	Responsivity	Mechanism	Polarization ratio	Work condition	Wavelength	Ref.
BP	0.35 mA/W	PTE effect	3.5	V_{sd} = 0 V	1700 nm	5
BP p-n (vertical)	1.5 mA/W	PN junction	35	V_{sd} = 0.1 V<br>V_g = 2.5 V	1700 nm	5
BP-antenna	14.2 mA/W	PTE effect	8.7	V_{sd} = 0 V	1550 nm	6
BP/WSe_2	0.5 A/W	Heterojunction	5.88	V_{ds} = 0.5 V	1550 nm	7
BP/InSe	11.7 mA/W	Heterojunction	10.76	V_{sd} = 0 V<br>V_{bg} = 0 V	633 nm	8
BP/MoS_2	0.9 A/W	Heterojunction	>100	V_{sd} = 0 V	3.6 um	9
Graphene/$PdSe_2$/Ge	691.5 mA/W	Heterojunction	112.2	V_{sd} = 0 V	650 nm	10
NbS_3	24.7 mA/W	Anisotropic material	1.84	V_{sd} = 0 V	830 nm	11
$ReSe_2$	1.5 mA/W	Anisotropic material	2	V_{sd} = 1 V	633 nm	12
TISe	1.48 A/W	Anisotropic material	2.56	V_{sd} = 1 V	633 nm	13
$ZrGeTe_4$	5.24 mA/W	Nanowire	1.31	V_{sd} = 0 V	1550 nm	14
InP	0.96 A/W	Nanowire	21	V_{sd} = 3 V	740 nm	15
CdSe	0.3 A/W	Nanowire	1.13	V_{sd} =35 V	488 nm	16
$CH_3NH_3PbI_3$	450 mA/W	Nanowire	1.3	V_{sd} = 1 V	530 nm	17
**BP homojunction**	**1.06 A/W**	**PV effect<br>PTE effect**	**288**	**V_{sd} = 0 V**	**1450 nm**	**This Work**

*PTE represents photothermoelectric effect and PV represents photovoltaic effect.

Supplementary References

1. Rodriguez B. J., Callahan C., Kalinin S. V. & Proksch R. Dual-frequency resonance-tracking atomic force microscopy. *Nanotechnology* **18**, 475504 (2007).
2. Gruverman A., Alexe M. & Meier D. Piezoresponse force microscopy and nanoferroic phenomena. *Nat. Commun.* **10**, 1661 (2019).
3. Xiang D. et al. Surface transfer doping induced effective modulation on ambipolar characteristics of few-layer black phosphorus. *Nat. Commun.* **6**, 6485 (2015).
4. Wang J., Jiang Y. & Hu Z. Dual-band and polarization-independent infrared absorber based on two-dimensional black phosphorus metamaterials. *Opt. Express* **25**, 22149-22157 (2017).
5. Yuan H. et al. Polarization-sensitive broadband photodetector using a black phosphorus vertical p-n junction. *Nat. Nanotechnol.* **10**, 707-713 (2015).
6. Venuthurumilli P. K., Ye P. D. & Xu X. Plasmonic resonance enhanced polarization-sensitive photodetection by black phosphorus in near infrared. *ACS Nano* **12**, 4861-4867 (2018).
7. Ye L. et al. Highly polarization sensitive infrared photodetector based on black phosphorus-on-WSe_2 photogate vertical heterostructure. *Nano Energy* **37**, 53-60 (2017).
8. Zhao S. et al. Highly polarized and fast photoresponse of black phosphorus-InSe vertical p-n heterojunctions. *Adv. Funct. Mater.* **28**, 1802011 (2018).
9. Bullock J. et al. Polarization-resolved black phosphorus/molybdenum disulfide mid-wave infrared photodiodes with high detectivity at room temperature. *Nat. Photon.* **12**, 601-607 (2018).
10. Wu D. et al. Highly polarization-sensitive, broadband, self-powered photodetector based on graphene/$PdSe_2$/germanium heterojunction. *ACS Nano* **13**, 9907-9917 (2019).
11. Wang Y. et al. Air-stable low-symmetry narrow-bandgap 2D sulfide niobium for polarization photodetection. *Adv. Mater.* **32**, e2005037 (2020).
12. Zhang E. et al. Tunable ambipolar polarization-sensitive photodetectors based on high-anisotropy $ReSe_2$ nanosheets. *ACS Nano* **10**, 8067-8077 (2016).
13. Yang S. et al. In-plane optical anisotropy and linear dichroism in low-symmetry layered TlSe. *ACS Nano* **12**, 8798-8807 (2018).
14. Bai R. et al. Polarization-sensitive and wide-spectrum photovoltaic detector based on quasi-1D $ZrGeTe_4$ nanoribbon. *InfoMat.* **4**, e12258 (2021).
15. Wang J., Gudiksen M. S., Duan X., Cui Y. & Lieber C. M. Highly polarized photoluminescence and photodetection from single indium phosphide nanowires. *Science* **293**, 1455-1457 (2001).
16. Singh A. et al. Polarization-sensitive nanowire photodetectors based on solution-synthesized CdSe quantum-wire solids. *Nano Lett.* **7**, 2999-3006 (2007).
17. Gao L. et al. Passivated single-crystalline $CH_3NH_3PbI_3$ nanowire photodetector with high detectivity and polarization sensitivity. *Nano Lett.* **16**, 7446-7454 (2016).
